# Red blood cell folate and depression risk in U.S. women: A dose–response analysis from a nationwide cross-sectional study (2009–2018)

**DOI:** 10.1097/MD.0000000000048023

**Published:** 2026-03-13

**Authors:** Weiqing Zeng, Hailiang Ma, Shanming Wei, Hongmei Li, Ziqi Jin, Liangji Zhou, Sheng Chai, Yongxing Tan, Gangjian Tang, Hua Wei

**Affiliations:** a Department of Orthopedics, Guilin Municipal Hospital of Traditional Chinese Medicine, Guilin, Guangxi, China; b Graduate College, Guangxi University of Chinese Medicine, Nanning, Guangxi, China; c Faculty of Chinese Medicine Science Guangxi University of Chinese Medicine, Nanning, Guangxi, China; d Department of Blood Transfusion, Suining Central Hospital, Suiningi, Sichuan, China.

**Keywords:** a cross-sectional study, depression, National Health and Nutrition Examination Survey, red blood cell folate, vitamin B9

## Abstract

In contemporary society, the prevalence of depression among women is worsening and rising annually, influenced by numerous intricate factors. Folate, a B vitamin, is vital for overall human health. This study aimed to investigate the association between red blood cell (RBC) folate levels and depression within a nationally representative group of women in the U.S. We conducted a cross-sectional study to examine the association between depression and RBC folate levels among women in the U.S., using data from the National Health and Nutrition Examination Survey from 2009 to 2018. Participants were stratified by depressive status and categorized into 4 quartiles (Q1–Q4) based on their RBC folate levels. We built both univariate and multivariate regression models to conduct a comprehensive threshold effect analysis on the correlation between RBC folate levels and depression. We analyzed data from 9409 women in the U.S. In the fully adjusted model, when RBC folate was sorted into 4 quartiles, both the lowest (Q1: odds ratio [OR] = 1.396, 95% confidence interval [CI]: 1.135–1.717, *P* = .0027) and highest (Q4: OR = 1.425, 95% CI: 1.160–1.749, *P* = .0014) quartiles showed significantly higher odds of depression compared to the reference Q2. Threshold effect analysis identified a breakpoint at 985 nmol/L, indicating a U-shaped association between RBC folate level and depression. Below 985 nmol/L, the risk of depression significantly decreased by 6% per 100 nmol/L increase in RBC folate (OR = 0.9994, 95% CI: 0.9989–0.9999, *P* = .0099). Above this inflection point, the risk significantly increased by 3% per 100 nmol/L increase (OR = 1.0003, 95% CI: 1.0002–1.0005, *P* < .0001). A U-shaped association was observed between RBC folate and depression among women in the U.S. Maintaining appropriate RBC folate levels may help reduce depression in women. However, the mechanisms behind both RBC folate and depression still require further in-depth research.

## 1. Introduction

Depression, particularly major depressive disorder, has emerged as a serious global public health crisis. A key issue is the rising prevalence and significant socio-economic impacts of this condition.^[1]^ Recent epidemiological studies reveal that from 1990 to 2019, the global incidence of depression surged by 59.3%, affecting approximately 290 million individuals.^[2]^ The COVID-19 pandemic made the situation worse. There was a 27.6% increase in depression prevalence and a 25.6% rise in anxiety disorders, highlighting how vulnerable mental health is to socio-environmental disruptions.^[1]^ Despite a slight decline (‐2.53%) in age-standardized incidence rates during this period due to demographic shifts,^[3]^ disparities persist across genders, ages, and regions.^[4]^ Women, for example, are disproportionately affected. In 2019, the age-standardized incidence rates for women was 2922.07 per 100,000, compared to 1688.81 for men.^[5]^ Depression is the second-leading cause of global disability-adjusted life years (DALYs), accounting for 12% of nonfatal health losses.^[6]^ In 2019, there were 5.78 million depression-related DALYs.^[7]^ High-income countries face a greater direct burden,^[3]^ while low-income regions struggle with wider treatment gaps due to limited healthcare resources.^[4]^ Notably, depression indirectly contributes to 16 million suicide-related DALYs and 4 million ischemic heart disease-related DALYs, showing its systemic health consequences.^[8]^ Economically, it reduces workforce productivity and increases healthcare costs, hindering global economic stability.^[9]^

Red blood cell (RBC) folate, a core biomarker for long-term folate storage, is crucial for deoxyribo nucleic acid synthesis, methylation metabolism, and erythropoiesis.^[10]^ It is considered the “gold standard” for assessing long-term folate status, as it reflects about 120 days of cumulative intake.^[11]^ RBC folate has dual clinical functions. It can improve erythropoietin resistance in chronic kidney disease, but paradoxically, it can increase cardiovascular mortality in hypertensive hyperhomocysteinemia. It also helps balance the efficacy and toxicity of methotrexate in pediatric leukaemia. Thus, context-specific monitoring and personalized management are needed.^[12–14]^ With the global implementation of folic acid fortification, measuring RBC folate is vital for disease diagnosis, treatment monitoring, and public health interventions.^[11]^ However, both folate deficiency and excess pose risks, requiring careful evaluation.^[15]^ The association between RBC folate and depression is still debated in the scientific community. Accordingly, our aim in this study was to assess the correlation between RBC folate and the likelihood of depression in adult U.S. females.

## 2. Materials and methods

### 2.1. Study population

**Figure 1.**
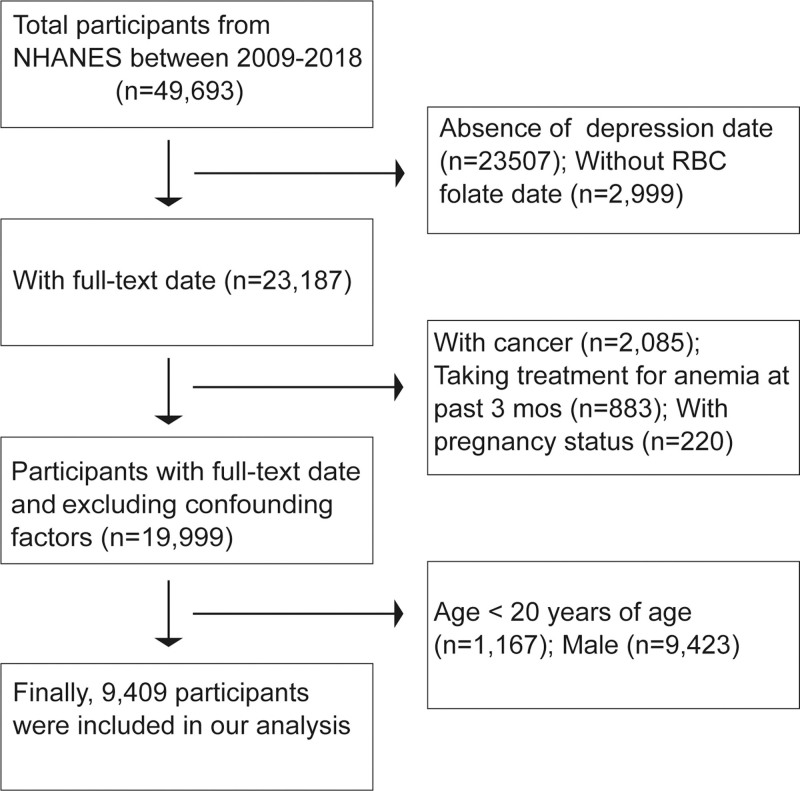
Flowchart of sample selection from the NHANES 2009 to 2018. NHANES = National Health and Nutrition Examination Survey.

The National Health and Nutrition Examination Survey (NHANES) is a valuable resource that provides comprehensive health and nutrition data for the U.S. population. This dataset serves as a robust analytical foundation for this study, enabling a deep exploration and understanding of the impact of various health and nutrition factors on individuals. The insights derived from NHANES not only enhance public health awareness but also provide a scientific basis for developing more effective health plans and services, thoroughly determining the prevalence of key health conditions and their risk factors in the community. For this study, we utilized NHANES data from 2009 to 2018 to guarantee both comprehensiveness and timeliness. Every participant gave their informed consent for the use of their anonymized data, and all methods were authorized by the National Health Statistics Research Center’s Ethics Review Committee. Additionally, due to the rigorous de-identification process of the dataset, the author’s institution’s institutional review board concluded that this work satisfied the exemption requirements. This study strictly adhered to the ethical guidelines of the Helsinki Declaration. In our study, participants had to be women aged 20 years or older. During the period from 2009 to 2018, the NHANES database provided 9409 eligible patients for our research. Among the 49,693 participants in the survey, 23,507 were excluded due to the absence of depression data, and an additional 2999 were excluded because their RBC folate status was missing. We excluded individuals who had cancer (n = 2085) or had been in therapy for anemia in the previous 3 months (n = 883). Additionally, we also excluded participants with pregnancy status (n = 220) or under the age of 20 (n = 1167). After excluding 9423 males, our analysis included data from 9409 participants finally (Fig. 1). The official website at www.cdc.gov/nchs/nhanes/ provides a summary of the exact methods used to gather data for the NHANES study. Whereas demographic data, health, and lifestyle were gathered through in-home interviews, the Mobile Examination Center was used for physical and medical examinations as well as the collection of blood and urine samples.

### 2.2. Depression status

During individual assessments at mobile examination centers, professionally trained interviewers used the 9-item Patient Health Questionnaire (PHQ-9) to identify signs of depressive symptoms. Each PHQ-9 item received a score between 0 and 3 (from “not at all” to “nearly every day”), giving a total score of 0 to 27. Depression was indicated by a PHQ-9 total score of ≥10, aligning with the moderate and severe depression categories.^[16,17]^

### 2.3. Folate status

From 2009 to 2018, the NHANES cycle implemented a multi-stage evaluation of the population’s folate status. In the 2009 to 2010 NHANES, the assessment primarily relied on microbiologic assay to assess folate levels in whole blood and serum, subsequently estimating RBC folate levels. However, during the NHANES evaluation cycle spanning from 2011 to 2018, the methodology was refined through the integration of 2 more precise analytical methods: liquid chromatography–tandem mass spectrometry with isotope dilution was employed to evaluate serum folate concentrations, whereas microbiologic testing was still used for measuring whole-blood folate levels. By synergistically applying these dual methodologies, the concentration of folate within red blood cells was more accurately derived. The computational formula employed is as follows: RBC folate concentration = {whole blood folate ‐ [serum blood folate * (1.0 ‐ hematocrit/100)]}/(hematocrit/100).

### 2.4. Covariates

The NHANES database supplied detailed data for the analysis of potential confounding factors. Blood urea nitrogen, serum iron, total protein, vitamin D, total cholesterol, serum creatinine, calcium, total bilirubin, and poverty income ratio were examples of continuous variables. The categorical variables included age (split into 3 age groups: 20–39, 40–59, and 60–80 years), body mass index (BMI), race, vigorous work activity, cohabitation status, educational attainment, alcohol intake (defined as at least 3 drinking experiences over the past 12 mos), and smoking status (defined as 100 or more cigarettes smoked in a lifetime). According to WHO guidelines, BMI was classified as “normal” (<25 kg/m^2^), “overweight” (25 kg/m^2^ ≤ BMI < 30 kg/m^2^), and “obese” (more than 30 kg/m^2^). For continuous variables with missing values, we used mean substitution. For categorical variables, we created a separate category for those with many missing values and merged those with few missing values into an “other” category. Detailed information on missingness is provided in Table S3, Supplemental Digital Content, https://links.lww.com/MD/R535. For more detailed information on the collection methods and specific details regarding RBC folate, depression, and other variables, it is recommended to visit the NHANES website (http://www.cdc.gov/nchs/nhanes/).

### 2.5. Statistical analysis

The association between RBC folate and depression (PHQ-9 ≥ 10) was examined without weighted multivariable logistic regression, and results are presented as odds ratios (ORs) with 95% confidence intervals (CIs). RBC folate was analyzed both as a continuous variable and in quartiles (Q1–Q4, with Q2 as the reference). Continuous variables were compared across quartiles without weighted linear regression, and categorical variables were compared with weighted Rao-Scott χ^2^ tests. The Strengthening the Reporting of Observational Studies in Epidemiology guidelines were followed while building regression models.^[18]^ Three models for regression were created: Model 2 had adjustments for age and race; all other variables were included in Model 3, and Model 1 was left unadjusted. To find potential inflection points, a piecewise linear regression model was used. Based on a number of variables, interaction and subgroup analyses were carried out in order to confirm sensitivity and look into possible moderating factors. Moreover, we performed internal validation using bootstrap resampling (1000 iterations with replacement) to confirm the stability of the results. Following NCHES analytic guidelines, we accounted for the complex survey design to minimize bias arising from selection, oversampling and nonresponse. Five 2-year cycles (2009–2018) were pooled, and the 2-year fasting-laboratory weight (WTMEC2YR) was scaled by 1/5 to create a 10-year weight appropriate for the biomarker subsample in which RBC folate was measured. Strata (SDMVSTRA) and primary sampling units (SDMVPSU) were specified in a single survey-set statement and used in Table 1 to ensure national representativeness, whereas all regression models were conducted without survey weights. All statistical analyses were conducted using R (version 3.4.3; R Foundation for Statistical Computing, Vienna, Austria) and EmpowerStats (version 5.0; Triad Scientific Solutions, Cary), with a significance level of *P* < .05.

**Table 1:** Basic characteristics of 9409 participants.

Characteristics	PHQ-9 < 10	PHQ-9 ≥ 10	*P*-value
	(n = 8372)	(n = 1037)	
Serum vitamin D (nmol/L)	72.20 ± 30.75	67.03 ± 29.48	<.0001
The ratio of income to poverty	2.96 ± 1.59	2.08 ± 1.51	<.0001
RBC folate (nmol/L)	1212.12 ± 526.44	1244.70 ± 715.83	.0837
Blood urea nitrogen (mmol/L)	4.58 ± 1.75	4.38 ± 1.85	.0010
Calcium (mmol/L)	2.34 ± 0.09	2.34 ± 0.09	.5685
Serum cholesterol (mmol/L)	5.03 ± 1.02	5.10 ± 1.13	.0456
Serum creatinine (µmol/L)	67.67 ± 18.69	70.12 ± 29.38	.0004
Serum iron (µmol/L)	14.40 ± 6.14	13.96 ± 6.06	.0396
Total bilirubin (µmol/L)	9.77 ± 4.47	9.06 ± 4.27	<.0001
Total protein (g/L)	70.69 ± 4.38	70.58 ± 4.57	.4615
Race (%)			.0582
Non-Hispanic White	65.37	62.12	
Non-Hispanic Black	11.51	12.53	
Mexican American	8.55	7.87	
Other race	14.57	17.49	
Living with partner (%)			<.0001
Yes	61.58	44.31	
No	38.37	55.66	
Missing	0.04	0.04	
Alcohol intake (%)			<.0001
Yes	17.88	24.93	
No	55.11	41.11	
Missing	27.01	33.96	
Vigorous work activity (%)			<.0001
Yes	13.29	18.47	
No	86.71	81.53	
Smoking status (%)			<.0001
Yes	34.67	55.65	
No	65.26	44.35	
Missing	0.06		
Age (%)			.0417
20 to 39 yr	38.23	37.30	
40 to 59 yr	37.32	41.13	
60 to 80 yr	24.45	21.57	
BMI (kg/m^2^) (%)			<.0001
<25	33.42	23.57	
≥25, <30	28.64	22.30	
≥30	37.94	54.13	

Mean ± SD for continuous variables: *P* value was calculated by the weighted linear regression model. % for categorical variables: *P* value was calculated by the weighted Chi-square test.

BMI = body mass index, PHQ-9 = 9-item Patient Health Questionnaire.

## 3. Results

### 3.1. Participants’ characteristics

The fundamental characteristics of the 9409 individuals in the current investigation are presented in Table 1. The individuals’ baseline features were stratified by depressive status. Individuals with vigorous work activity, smokers, alcohol intake, living without a partner, 40 to 59 years of age, and BMI ≥30 were more likely to suffer from depressive disorders. They also exhibited lower levels of blood urea nitrogen, poverty income ratio, serum vitamin D, serum iron, and total bilirubin than those without depression.

### 3.2. Associations of RBC folate with depression

Table 2 illustrates that we developed 3 multivariate regression models to explore the independent relationship between RBC folate and the likelihood of depression, with RBC folate categorized into 4 quartiles. Participants in the lowest folate quartile (Q1) had 39.6% greater odds of reporting depression compared to those in the reference quartile (Q2) (OR = 1.3957, 95% CI: 1.1346, 1.7168, *P* = .0027). Similarly, the highest group (Q4) was 42.5% higher than the reference group (OR = 1.4246, 95% CI: 1.1602, 1.7492, *P* = .0014). The need for more investigation into the underlying processes and clinical consequences is highlighted by these findings, which suggest that women may be more susceptible to depression if their RBC folate levels are high or low.

**Table 2:** Association of RBC folate with depression and quartiles of RBC folate in U.S. women.

RBC folate (nmol/L) quartile	Model 1 OR (95% CI) *P*-value	Model 2 OR (95% CI) *P*-value	Model 3 OR (95% CI) *P*-value
Q2 (114–793)	Ref.	Ref.	Ref.
Q1 (794–1020)	1.4868 (1.2097–1.8274) .0003	1.4984 (1.2280–1.8285) .0002	1.3957 (1.1346–1.7168) .0027
Q3 (1030–1330)	1.1531 (0.9274–1.4338) .2042	1.1477 (0.9227–1.4275) .2199	1.2479 (1.0048–1.5497) .0507
Q4 (1340–6750)	1.2746 (1.0334–1.5722) .0265	1.2311 (1.0086–1.5026) .0444	1.4246 (1.1602–1.7492) .0014
*P* for trend	.26	.38	.51

Model 1: no covariates were adjusted.

Model 2: age, race were adjusted.

Model 3: age, race, the ratio of income to poverty, serum iron, calcium, vitamin D, total protein, total cholesterol, blood urea nitrogen, serum creatinine, living with partner, total bilirubin, smoking at least 100 cigarettes in life, vigorous work activity, educational level, drinking at least 3 alcohol over past 12 mos and BMI were adjusted.

BMI = body mass index, OR = odds ratio, RBC = red blood cell.

### 3.3. Nonlinear relationships

**Figure 2.**
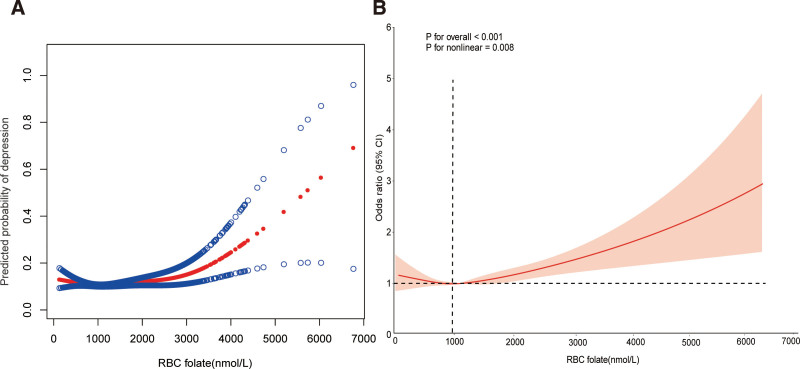
The smooth curve fitting illustrated the association between RBC folate level and depression. RBC = red blood cell.

Using smooth curve fittings (Fig. 2), we examined the potential nonlinear association between RBC folate levels and depression. We observed a U-shaped relationship between RBC folate level and depression in Table 3, with significant linear patterns and inflection points of 985 nmol/L RBC folate. In individuals with RBC folate levels below 985 nmol/L, a 6% reduction in the incidence of depression is linked to every 100 nmol/L rise in RBC folate (OR = 0.9994, 95% CI: 0.9989, 0.9999, *P* = .0099). In contrast, the risk of depression increases by 3% for every 100 nmol/L rise in folate levels when RBC folate levels are above 985 nmol/L (OR = 1.0003, 95% CI: 1.0002, 1.0005, *P* < .0001). These findings indicate a statistically significant nonlinear relationship between RBC folate levels and the odds of depression, as demonstrated by the linear regression analysis.

**Table 3:** Threshold effect analysis between RBC folate and depression in U.S. women.

	Adjusted OR (95% CI), *P*-value
RBC folate (nmol/L)	
Fitting by standard linear model	1.0002 (1.0000–1.0003) .0112
Fitting by two-piecewise linear model	
(K) inflection point	985
RBC folate < 985 (nmol/L)	0.9994 (0.9989–0.9999) .0099
RBC folate > 985 (nmol/L)	1.0003 (1.0002–1.0005) <.0001
Log-likelihood ratio	0.015

Age, race, living with parter, the ratio of income to poverty, serum iron, calcium, vitamin D, total protein, total cholesterol, blood urea nitrogen, serum creatinine, total bilirubin, smoking at least 100 cigarettes in life, vigorous work activity, educational level, drinking at least 3 alcohol over past 12 mos and BMI were adjusted.

BMI = body mass index, OR = odds ratio, RBC = red blood cell.

### 3.4. Subgroup analyses

To verify the sensitivity and consistency of the correlation between RBC folate and depression across different subgroups, we conducted stratified subgroup and interaction analyses based on various variables, as presented in Table 4. We found that this association was consistent across subgroups of age, cohabitation status, educational attainment, alcohol intake, smoking status, vigorous work activity, and race (all *P* for interaction > .05). In addition, we found that variables of BMI may have interactions with RBC folate associated with depression (*P* for interaction < .05).

**Table 4:** Subgroup analysis of association between RBC folate and depression among U.S. women.

Subgroups	n	OR (95% CI, *P*-value)	*P* for interaction
BMI			.0024
BMI < 25	2783	0.9967 (0.9932–1.0001) .0599	
BMI ≥ 25, BMI < 30	2652	1.0008 (0.9978–1.0037) .6118	
BMI ≥ 30	3974	1.0032 (1.0015–1.0049) .0002	
Living with partner			.2854
Yes	5150	1.0025 (1.0005–1.0045) .0124	
No	4255	1.0011 (0.9993–1.0029) .2440	
Missing	4	0.8472 (0.0000–inf.) .9935	
Ages			.3592
20–39 yr	3353	1.0026 (0.9998–1.0055) .0729	
40–59 yr	3307	1.0029 (1.0008–1.0050) .0080	
60–80 yr	2749	1.0008 (0.9986–1.0030) .4889	
Race			.5077
Non-Hispanic White	3614	1.0024 (1.0006–1.0043) .0094	
Non-Hispanic Black	1981	1.0021 (0.9987–1.0055) .2267	
Mexican American	1460	1.0029 (0.9989–1.0070) .1602	
Other race	2354	0.9998 (0.9966–1.0029) .8943	
Educational level (%)			.3827
Less than some college	4037	1.0010 (0.9992–1.0028) .2837	
Some college	3090	1.0024 (1.0002–1.0046) .0362	
More than college	2275	1.0034 (0.9999–1.0070) .0566	
Missing	7	1.0056 (0.0000–inf.) .9998	
Vigorous work activity (%)			.6549
Yes	1177	1.0010 (0.9972–1.0047) .6206	
No	8232	1.0019 (1.0005–1.0033) .0096	
Smoking at least 100 cigarettes in life (%)			.2903
Yes	3211	1.0026 (1.0007–1.0044) .0077	
No	6192	1.0011 (0.9992–1.0030) .2433	
Missing	6	0.9901 (0.0000–inf.) .9995	
Drinking at least 3 alcohol over past 12 mos (%)			.5880
Yes	1598	1.0027 (0.9992–1.0062).1311	
No	4454	1.0023 (1.0001–1.0044).0377	
Missing	3357	1.0010 (0.9991–1.0029) .2961	

Subgroup analysis for the association between RBC folate (nmol/dL) and depression. Each stratification was adjusted for covariates listed in Table 1 except for the stratifying variable itself.

OR = odds ratio, RBC = red blood cell.

### 3.5. Bootstrap validation

To evaluate reproducibility, we repeated the analyses in 1000 bootstrap samples drawn with replacement. The continuous RBC-folate OR remained 1.0003 (95% CI 1.0000–1.0006; *P* = .012), and across quartiles Q1, Q3, and Q4, the respective ORs were 1.3980 (1.134–1.720; *P* = .002), 1.2501 (1.004–1.549; *P* = .047), and 1.4265 (1.160–1.749; *P* = .001) versus Q2 in Table 5, with no linear trend (*P* = .52). Likewise, in Table 6, the two-segment model anchored at 985 nmol/L reproduced the original inflection: OR 0.9995 (0.9990–1.0000; *P* = .010) below and 1.0004 (1.0001–1.0006; *P* < .001) above the threshold, while the log-likelihood ratio test retained significance (*P* = .018), confirming the robustness of the observed breakpoint.

**Table 5:** Bootstrap validation of quartile-based associations between RBC folate and depression in U.S. women (reference: Q2).

RBC folate (nmol/L) quartile	Model 3 OR (95% CI) *P*-value	Bootstrap OR (95% CI) *P*-value
Q2 (114–793)	Ref.	Ref.
Q1 (794–1020)	1.3957 (1.1346–1.7168) .0027	1.3980 (1.1340–1.7200) .002
Q3 (1030–1330)	1.2479 (1.0048–1.5497) .0507	1.2501 (1.0040–1.5490) .047
Q4 (1340–6750)	1.4246 (1.1602–1.7492) .0014	1.4265 (1.1600–1.7490) .001
*P* for trend	0.51	0.52

Bootstrap based on 1000 nonparametric resamples with replacement from the analytic sample; estimates adjusted for age, race, the ratio of income to poverty, serum iron, calcium, vitamin D, total protein, total cholesterol, blood urea nitrogen, serum creatinine, living with partner, total bilirubin, smoking at least 100 cigarettes in life, vigorous work activity, educational level, drinking at least 3 alcohol over past 12 mos and BMI were adjusted.

BMI = body mass index, OR = odds ratio, RBC = red blood cell.

**Table 6:** Bootstrap validation of threshold effect analysis between RBC folate and depression among U.S. women.

	Adjusted OR (95% CI), *P*-value	Bootstrap OR (95% CI) *P*-value
RBC folate (nmol/L)		
Fitting by standard linear model	1.0002 (1.0000–1.0003) .0112	1.0003 (1.0000–1.0006) .012
Fitting by two-piecewise linear model		
RBC folate < 985 (nmol/L)	0.9994 (0.9989–0.9999) .0099	0.9995 (0.9990–1.0000) .010
RBC folate > 985 (nmol/L)	1.0003 (1.0002–1.0005) <.0001	1.0004 (1.0001–1.0006) <.001
Log-likelihood ratio	0.015	0.018

Bootstrap resampling was performed with 1000 iterations. Estimates adjusted for age, race, living with parter, the ratio of income to poverty, serum iron, calcium, vitamin D, total protein, total cholesterol, blood urea nitrogen, serum creatinine, total bilirubin, smoking at least 100 cigarettes in life, vigorous work activity, educational level, drinking at least 3 alcohol over past 12 mos and BMI.

BMI = body mass index, OR = odds ratio, RBC = red blood cell.

### 3.6. Sensitivity analysis

To enhance methodological rigour, we performed a complete-case analysis excluding any participant with missing data on any covariate (final n = 5986; ~63.6% of the original analytic sample). Using the primary effect estimate (the adjusted OR per 1 nmol/L increase in RBC folate) we observed consistent directionality, similar point estimates, and statistical significance, confirming the robustness of our main finding. Furthermore, we conducted MI using the MICE algorithm (m = 20 imputations), incorporating all covariates and the outcome in the imputation model. The resulting estimate (OR = 1.0002, 95% CI: 1.0000–1.0004; *P* = .018) remained highly consistent with our primary analysis (Table S4, Supplemental Digital Content, https://links.lww.com/MD/R535).

## 4. Discussion

To our knowledge, this is the first study to reveal a U-shaped relationship between RBC folate and depression among women in the U.S. Our results indicate a nonlinear correlation between depression and RBC folate levels at 985 nmol/L. This implies that the effect of RBC folate on depression varies with its concentration. These findings emphasize the importance of maintaining adequate RBC folate levels for mental health. They imply that women with RBC folate levels below 985 nmol/L may benefit from treatments that increase folate consumption. On the contrary, taking folate supplements may have an adverse effect when RBC folate levels are excessive.

Previous studies have explored the relationship between depression and RBC folate levels in different populations. In individuals aged 60 and older, those in the lowest 20% of RBC folate status had a higher risk of depression.^[19]^ However, Nguyen et al found no statistically significant difference in median RBC folate contents between depressed and nondepressed women (*P* = .2).^[20]^ Even after accounting for other covariates, people with a lifelong diagnosis of severe depression or dysthymia had lower RBC folate levels than those who had never been depressed.^[21]^ Nondepressed hemodialysis patients had significantly higher mean RBC folate levels than depressed ones.^[22]^ Ramos et al discovered that as RBC folate content increased, the relative odds of dementia and cognitive impairment reduced.^[23]^ In Guatemalan women, low RBC folate was linked to higher baseline depressive symptoms.^[24]^ Hintikka et al reported a slightly positive relationship between the degree of depression and folate levels.^[25]^ Yang et al found a significant negative correlation between RBC folate levels and severe depression.^[26]^ These conflicting findings indicate that the relationship between RBC folate and depression is complex and may vary with the study population and measurement methods. Future research is needed to further explore the mechanisms behind these associations.

The exact biological processes behind the association between depression and RBC folate levels, which involves a biphasic relationship with threshold-dependent mechanisms, remain unknown. Emerging evidence suggests a nonlinear, biphasic relationship between RBC folate levels and the risk of depression in women, with a critical threshold at around 985 nmol/L. Below this threshold, folate supplementation may help relieve depressive symptoms. However, excessive supplementation above this level can unexpectedly increase the risk of depression. This dual effect is mediated by folate’s multifaceted roles in neurobiological pathways, which are influenced by baseline nutritional status, genetic polymorphisms, and interactions with inflammatory and neurotransmitter systems.^[27–29]^

Low RBC folate levels may influence depression via multiple mechanisms, and folate supplementation may offer antidepressant benefits. Low RBC folate disrupts S-adenosyl methionine (SAMe) production, impairing monoamine synthesis and synaptic plasticity.^[30–32]^ Women with the thymine–thymine genotype may have lower SAMe levels and a higher risk of depression.^[33,34]^ In folate-deficient states, folate supplementation restores normal SAMe levels, boosts neurotransmitter availability and receptor methylation, and improves mood.^[35,36]^

On the other hand, high folate levels may have pro-depressive effects. Excessive folate can alter gut microbiota composition, reducing *Bifidobacterium* and *Lactobacillus* species that produce neuroactive metabolites. Gut microbiota dysbiosis can raise intestinal permeability, leading to higher systemic lipopolysaccharide levels, which can trigger neuroinflammation and depressive behavior.^[37,38]^ High-dose folate stimulates the body to produce autoantibodies against folate receptors (α/β) in the choroid plexus and hippocampus. These antibodies can block folate transport to the brain. Even with normal peripheral folate levels, they can cause cerebral folate deficiency, more common in women with late-onset depression.^[39]^

Overall, the research shows that both low and high RBC folate levels may induce depression. We believe this phenomenon warrants further investigation. We have analyzed potential reasons for the observed modest association, including the possibility that RBC folate could be a biomarker for other metabolic processes that are key to depression likelihood.^[40]^

This study had several advantages. The research report unveiled, for the first time, a U-shaped relationship between RBC folate levels and female depression. With a large sample size and national representativeness, it could more accurately mirror the overall situation. Additionally, the selected RBC folate index was more stable, and a smooth curve-fitting method was employed to explore potential nonlinear correlations. However, it is crucial to acknowledge the limitations. Firstly, although most possible confounding factors were included, we were unable to completely rule out the existence of other residual confounding factors. Secondly, due to the absence of serum total folate information in the 2009 to 2010 NHANES data, the study only used RBC folate to represent folate status. Thirdly, the study did not examine the severity of individual depression, which could result in certain statistical biases. Fourthly, the measurement method of RBC folate in the 2009 to 2010 NHANES differed from other studies, potentially affecting the determination of RBC folate levels, although all primary analyses are now presented separately for 2009 to 2010 and 2011 to 2018 and are contrasted with the pooled 2009 to 2018 estimates (see Tables S1 and S2, Supplemental Digital Content, https://links.lww.com/MD/R535). However, an additional association analysis between RBC folate levels and the likelihood of depression during the 2011 to 2018 NHANES cycle yielded consistent results. Fifthly, our multivariable and nonlinear regression models did not incorporate NHANES survey weights. While this decision helped avoid model divergence and yielded stable point estimates, it may compromise the national representativeness of our findings and the accuracy of standard error estimation. Sixthly, folate status can be influenced by various factors such as diet, supplement use, and genetics, and can vary significantly over an individual’s life.^[41,42]^ Similarly, as a chronic disease, depression can progress over time, with stable and worsening phases.^[43]^ Yet, the cross-sectional design of this study could not capture the dynamic fluctuations in folate levels and the progression of depression. Thus, this study provides only a cross-sectional view and may not fully represent the complex interactions between these 2 variables.

## 5. Conclusion

A U-shaped association was observed between RBC folate and depression among women in the U.S. Maintaining appropriate RBC folate levels may help reduce depression in women. However, the mechanisms of action of both still require further in-depth research.

## Acknowledgments

The authors appreciate the NHANES research participants and staff for their valuable contributions, as well as to the supporting funding projects.

## Author contributions

**Conceptualization:** Weiqing Zeng, Hua Wei.

**Data curation:** Hongmei Li, Hua Wei.

**Software:** Ziqi Jin, Sheng Chai, Yongxing Tan.

**Validation:** Liangji Zhou, Gangjian Tang.

**Writing – original draft:** Shanming Wei, Ziqi Jin.

**Writing – review & editing:** Weiqing Zeng, Hailiang Ma.

## Supplementary Material
