# Supplementary material for: Red blood cell folate and depression risk in U.S. women: A dose–response analysis from a nationwide cross-sectional study (2009–2018)
Source: Medicine (Baltimore). 2026 Mar 13;105(11):e48023. doi: 10.1097/MD.0000000000048023 (PMC12991464; doi:10.1097/MD.0000000000048023)
Supplement: Supplementary file 1 [file medi-105-e48023-s001.docx]

**Supplementary Table 1 Associations between RBC folate and depression in U.S. women, presented separately for 2009–2010 and 2011–2018 (reference: Q2)**

| **RBC folate(nmol/L)**  **quartile** | **Model 3 OR (95%CI) P-value (2009 - 2018)** | **Model 3 OR (95%CI) P-value (2009 - 2010)** | **Model 3 OR (95%CI) P-value (2011 - 2018)** |
| --- | --- | --- | --- |
| Q1 vs Q2 | 1.40 (1.13,1.72) 0.003 | 1.40 (1.09,1.80) 0.009 | 1.38 (1.11,1.72) 0.004 |
| Q4 vs Q2 | 1.42 (1.16,1.75) 0.001 | 1.45 (1.11,1.90) 0.007 | 1.41 (1.14,1.75) 0.002 |

Bootstrap based on 1000 non-parametric resamples with replacement from the analytic sample; estimates adjusted for age, race, the ratio of income to poverty, serum iron, calcium, vitamin D, total protein, total cholesterol, blood urea nitrogen, serum creatinine, living with parter, total bilirubin, smoking at least 100 cigarettes in life, vigorous work activity, educational level, drinking at least 3 alcohol over past 12 mos and BMI were adjusted.

**Supplementary Table 2 Threshold effect analysis between RBC folate and depression, presented separately for 2009–2010 and 2011–2018**

|  | **Adjusted OR (95% CI), p-value (2009 - 2018)** | **Adjusted OR (95% CI), p-value (2009 - 2010)** | **Adjusted OR (95% CI), p-value (2011 - 2018)** |
| --- | --- | --- | --- |
| RBC folate (nmol/L) |  |  |  |
| Fitting by standard linear model | 1.0002 (1.0000, 1.0003) 0.0112 | 1.0001 (0.9999, 1.0003) 0.18 | 1.0003 (1.0001, 1.0004) 0.003 |
| Fitting by two-piecewise linear model |  |  |  |
| (K)Inflection point | 985 | 985 | 985 |
| RBC folate < 985(nmol/L) | 0.9994 (0.9989, 0.9999) 0.0099 | 0.9992 (0.9985, 0.9999) 0.021 | 0.9995 (0.9991, 0.9999) 0.015 |
| RBC folate > 985 (nmol/L) | 1.0003 (1.0002, 1.0005) <0.0001 | 1.0002 (1.0000, 1.0004) 0.026 | 1.0004 (1.0002,1.0006), <0.001 |
| Log-likelihood ratio | 0.015 | 0.042 | 0.009 |

Age, race, living with parter, the ratio of income to poverty, serum iron, calcium, vitamin D, total protein, total cholesterol, blood urea nitrogen, serum creatinine, total bilirubin, smoking at least 100 cigarettes in life, vigorous work activity, educational level, drinking at least 3 alcohol over past 12 mos and BMI were adjusted.

**Supplementary Table 3** Missing Data Patterns for All Analytical Covariates (N = 9,409)

| **Variable** | **Type** | **Missing, n (%)** | **Handling Strategy** |
| --- | --- | --- | --- |
| Age group | Categorical | 0 (0.0) | — |
| Sex | Categorical | 0 (0.0) | — |
| Race/ethnicity | Categorical | 0 (0.0) | — |
| Body mass index (BMI) category | Categorical | 0 (0.0) | — |
| Educational attainment | Categorical | 7 (0.07) | “Missing” category created |
| Cohabitation status | Categorical | 4 (0.04) | “Missing” category created |
| Alcohol≥3 drinks in past 12 months) | Categorical | 3,357 (35.7) | “Missing” category retained; reported in subgroup analysis |
| Smoking status | Categorical | 0 (0.0) | — |
| Vigorous work activity | Categorical | 0 (0.0) | — |
| Poverty income ratio (PIR) | Continuous | 126 (1.3) | Mean imputation |
| Serum vitamin D (nmol/L) | Continuous | 8 (0.09) | Mean imputation |
| Serum iron (μg/dL) | Continuous | 42 (0.45) | Mean imputation |
| Total protein (g/dL) | Continuous | 31 (0.33) | Mean imputation |
| Blood urea nitrogen (mg/dL) | Continuous | 28 (0.30) | Mean imputation |
| Serum creatinine (mg/dL) | Continuous | 19 (0.20) | Mean imputation |
| Calcium (mg/dL) | Continuous | 35 (0.37) | Mean imputation |
| Total bilirubin (mg/dL) | Continuous | 22 (0.23) | Mean imputation |
| Total cholesterol (mg/dL) | Continuous | 53 (0.56) | Mean imputation |
| PHQ-9 ≥10 | Outcome | 0 (0.0) | — |
| RBC folate (nmol/L) | Exposure | 0 (0.0) | — |

All variables were derived from the NHANES 2009–2018 cycles. Missingness for alcohol use reflects NHANES sampling design (alcohol questionnaire administered only to a subsample). Continuous variables with <1% missingness were imputed using the overall sample mean. Categorical variables with minimal missing values (<0.1%) were assigned a separate “Missing” category to preserve sample size and avoid bias from arbitrary recoding.

**Supplementary Table 4**. Sensitivity Analyses of the Association Between RBC Folate and Depression Status Under Different Missing Data Handling Approaches

| **Analysis Method** | **Analytic Sample Size** | **Adjusted Odds Ratio per 1 nmol/L Increase in RBC Folate (95% CI)** | ***p*** Value |
| --- | --- | --- | --- |
| Primary analysis  (Mean imputation for continuous variables; ”Missing” categories for categorical variables) | 9,409 | 1.0002 (1.0000, 1.0003) | 0.011 |
| Complete-case analysis  (Excluding all observations with any missing covariate) | 5,986 | 1.0003 (1.0001, 1.0005) | 0.003 |
| Multiple imputation (MICE, m = 20)(All covariates + outcome included in imputation model) | 9,409 | 1.0002 (1.0000, 1.0004) |  |

All models were adjusted for age, sex, race/ethnicity, BMI category, education, cohabitation status, smoking, vigorous activity, PIR, serum vitamin D, iron, total protein, BUN, creatinine, calcium, bilirubin, and total cholesterol. The direction and magnitude of association remained consistent across all approaches, supporting the robustness of the primary finding. Multiple imputation was performed using the fully conditional specification (FCS) method with predictive mean matching for continuous variables and logistic regression for binary/categorical variables.
